# Lambl's Excrescences: An Enigma of Modern Diagnostic Cardiology

**DOI:** 10.7759/cureus.6407

**Published:** 2019-12-17

**Authors:** Hina Amin, Mohammad Hashim Jilani, Daniel Villarreal

**Affiliations:** 1 Internal Medicine, State University of New York Upstate Medical University, Syracuse, USA; 2 Cardiovascular Medicine, State University of New York Upstate Medical University, Syracuse, USA

**Keywords:** lambl's excrescences, echo-densities, cardio-embolic stroke, papillary fibroelastomas, valve strands, cardioembolic stroke

## Abstract

Lambl's excrescences were first described in 1856 by a Bohemian physician, Vilém Dušan Lambl, and since then have gained widespread attention and controversy within the medical literature. Despite numerous case reports and observational studies, consensus on the significance and management of Lambl’s excrescences remains sparse. We describe the case of a 48-year-old male who presented with recurrent embolic strokes. No underlying paroxysmal arrhythmia or inter-atrial shunt was identified, and the only pathological finding was a 1-mm aortic valve strand. We managed this patient successfully using a novel oral anticoagulant.

## Introduction

Embolic stroke is a result of thrombogenic material originating from cardiac, aortic, or, rarely, unidentified sources and dislodging into the cerebral vasculature. Cardioembolism accounts for roughly one-fourth of all ischemic strokes [1]. A rare etiology encountered in patients presenting with cardioembolic stroke are Lambl's excrescences (LEs), which are filiform extensions that form along the lines of valve closure due to the age-related degenerative process. LEs are primarily diagnosed through TEE (transesophageal echocardiogram) where they are visualized as mobile filamentous echo-densities on aortic, mitral, and prosthetic valves, as well as subvalvular structures. Structurally, they are composed of an acellular stroma covered by the endothelium. These valvular strands have the potential to cause thromboembolic complications such as acute coronary syndrome and ischemic stroke.

## Case presentation

**Figure 1:**
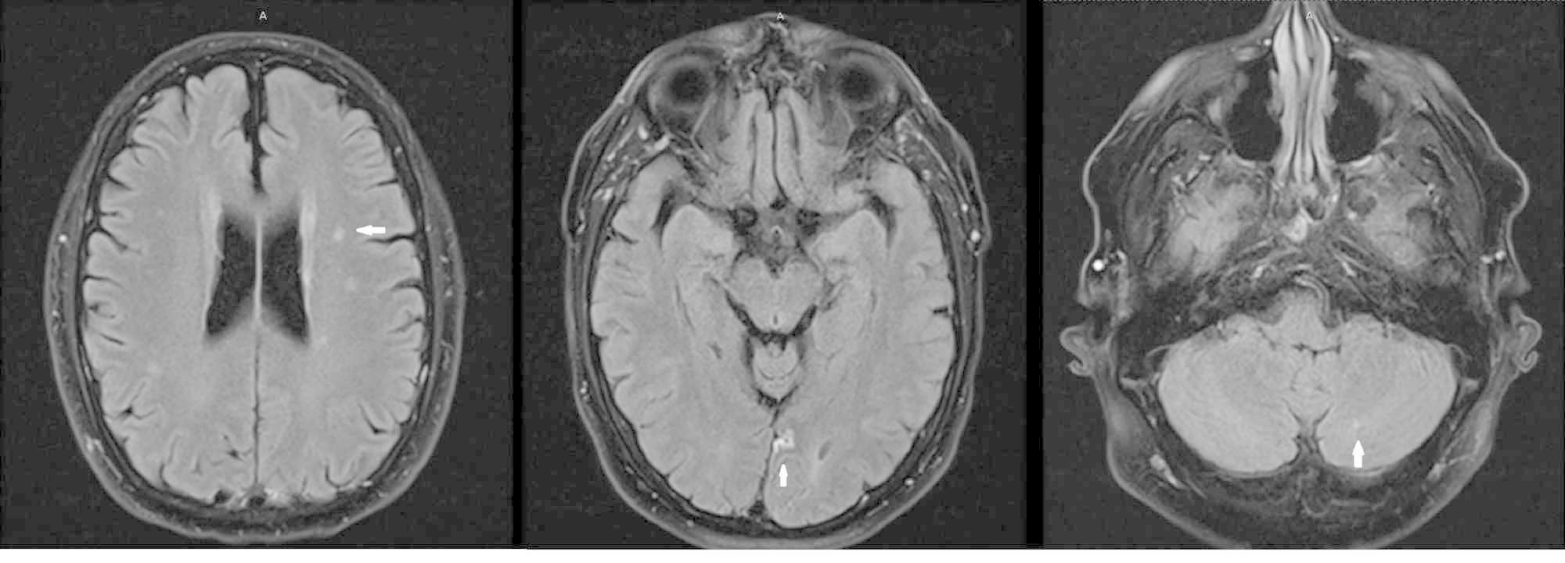
Brain MRI without contrast showing acute ischemic infarcts in the left thalamus (extreme left), the medial occipital lobe (middle), and the cerebellum (extreme right)

A 48-year-old hypertensive and diabetic male presented with a complaint of vertigo and vomiting. His medical history was significant for similar episodes of dizziness; however, workup did not reveal any cardiac or neurologic abnormality, and he was managed symptomatically. His physical examination was positive for bilateral horizontal nystagmus and his NIHSS (National Institutes of Health Stroke Scale) score was 0 [2]. Brain MRI showed multiple hypodensities in the left thalamic, the left occipital region, and the cerebellum, indicative of an embolic process (Figure 1).

**Figure 2:**
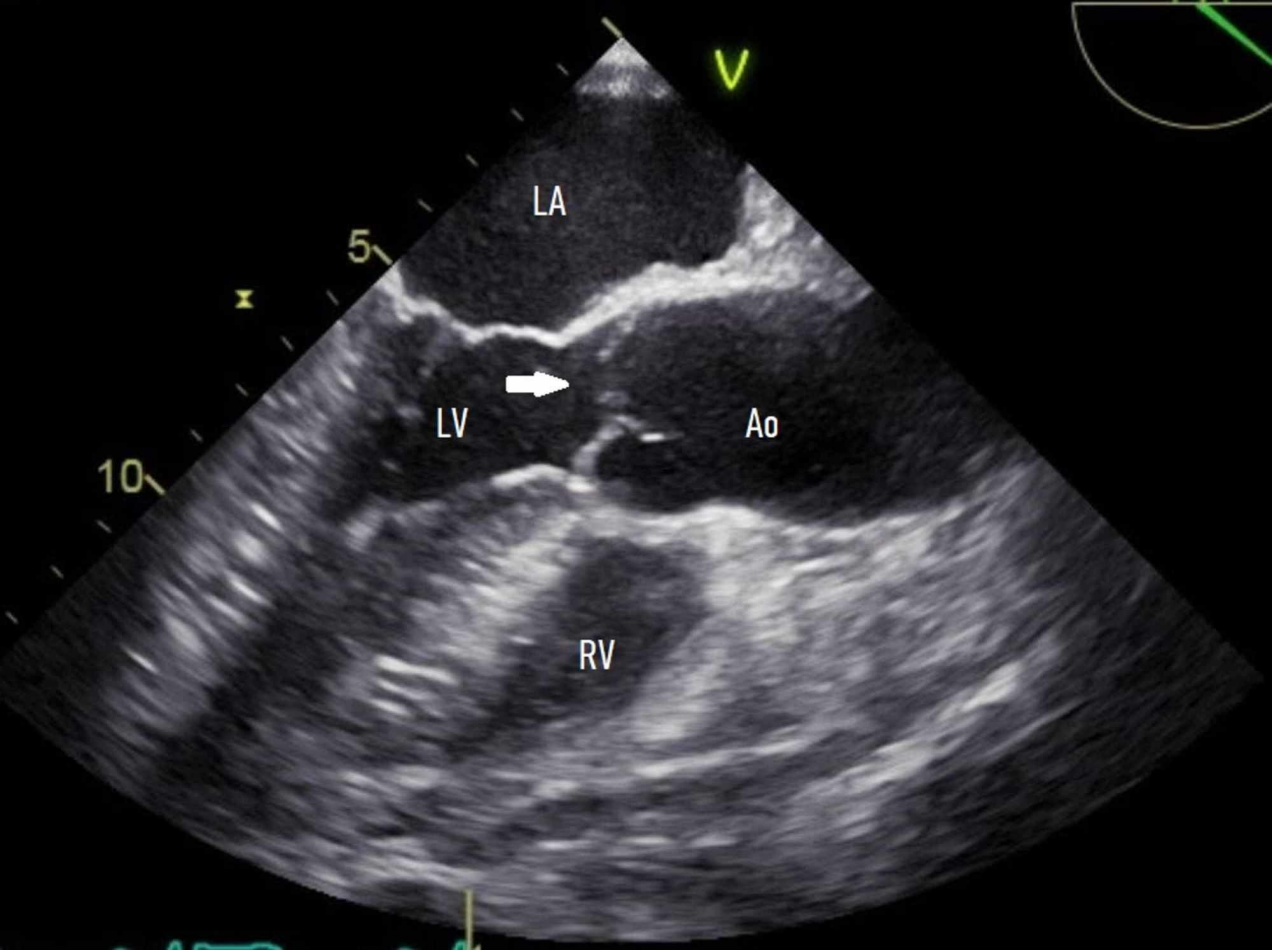
TEE, mid-esophageal long -axis view, showing an approximately 1 -mm echo-density on the ventricular side of the aortic valve (arrow) TEE, transesophageal echocardiogram; LA, left atrium; LV, left ventricle; RV, right ventricle; Ao, aortic root

The thyroid profile and hypercoagulability workup were unremarkable. Due to the symptom onset of >4.5 hours and NIHSS score of 0, thrombolysis was not attempted and he was started on aspirin and high-intensity statin. CT angiogram of the head and neck was negative for arterial occlusion, stenosis, or aneurysm. EKG and telemetry were negative for any arrhythmias. A two-dimensional echocardiogram (ECG) showed normal left ventricle size and function, normal left atrial size, and no valve abnormalities. TEE with a bubble study was then performed, which showed a single 1-mm echo- density on the ventricular side of the aortic valve, consistent with an LE (Figure 2).

A literature review of clinical guidelines in the management of LEs was completed. After consolidating all evidence, the decision was made to treat the patient with anticoagulation alone until a definitive cause of the stroke was isolated. Aspirin was stopped. A loop recorder inserted on discharge was negative for paroxysmal atrial arrhythmia. At three months, he was free of any new neurological events, and a decision was made to continue anticoagulation therapy.

## Discussion

Cardioembolic stroke secondary to LEs is a diagnosis of exclusion and has been subject to considerable controversy. A prospective analysis of lupus patients found a similar incidence of stroke in patients with and without LEs and a similar prevalence of LEs in patients with and without cerebrovascular disease [3]. On the contrary, several isolated case reports support at least some association between LEs and stroke, including one retrospective analysis by Freedberg et al., which showed a higher prevalence of LEs in patients with embolic events compared with patients without embolic events [4]. Similarly, other authors found a high incidence of strands in patients with cardioembolism in both native and prosthetic valves [5]. Due to conflicting evidence in the literature, it is a widespread consensus that valve strands carry an association with thromboembolism that is most likely noncausal in nature. For this association to be established, strands need to be differentiated from other valve pathologies, and other etiologies for cryptogenic stroke should simultaneously be ruled out. Literature on the management of LEs in patients with and without stroke is currently limited, and standard guidelines regarding the management of LEs are not well established. Management of these patients is largely based on anecdotal case reports and is most often individualized on a case-to-case basis. Asymptomatic LEs are mostly managed conservatively. While some authors resorted to surgical removal of the excrescences at the first episode of stroke, many others opted for anticoagulation or antiplatelet therapy with the initial occurrence of stroke and reserved surgical management only for a recurrent stroke. Both proclaimed treatment strategies were associated with a favorable outcome at three months to one year follow-up. Reportedly, large LEs (4-17 mm, rarely > 20 mm) are more likely to be associated with recurrent embolic strokes and thus warrant surgical intervention. Large LEs should be differentiated from papillary fibroelastomas (the most common benign cardiac neoplasm of cardiac valves), a diagnosis that merits surgical removal due to a high embolic risk associated with it. In our case, an exhaustive stroke workup did not reveal a definite etiology for the patient’s stroke; however, we discovered a 1-mm aortic valve LE. Since this was the first instance of embolic stroke associated with LEs, we decided to initiate anticoagulation using a novel oral anticoagulant after a risk-benefit discussion of operative versus medical management. Our patient remained free of recurrence of stroke with medical management alone, thus supporting this strategy for the symptomatic patient.

This article has been presented as an abstract at the Southern Regional Meeting 2019, New Orleans, LA, February 21-23, 2019 (Amin H, Jilani M, Pitroda P, Villarreal D. Lambl’s excrescences; an enigma of diagnostic cardiology; https://jim.bmj.com/content/67/2/354).

## Conclusions

Due to a lack of concise and agreeable treatment strategies, the management of LE is often at the discretion of the physician. We conclude that LE should be considered in the differential of cardioembolic stroke after excluding other etiologies. Anticoagulation should be continued for at least three months after initial implementation.
